# GIANT 2.0: genome-scale integrated analysis of gene networks in tissues

**DOI:** 10.1093/nar/gky408

**Published:** 2018-05-25

**Authors:** Aaron K Wong, Arjun Krishnan, Olga G Troyanskaya

**Affiliations:** 1 Center for Computational Biology, Flatiron Institute, Simons Foundation, New York, NY 10010, USA; 2 Department of Computational Mathematics, Science and Engineering, Michigan State University, East Lansing, MI 48824, USA; 3 Department of Biochemistry and Molecular Biology, Michigan State University, East Lansing, MI 48824, USA; 4 Department of Computer Science, Princeton University, Princeton, NJ 08544, USA; 5 Lewis-Sigler Institute for Integrative Genomics, Princeton University, Princeton, NJ 08544, USA

## Abstract

GIANT2 (Genome-wide Integrated Analysis of gene Networks in Tissues) is an interactive web server that enables biomedical researchers to analyze their proteins and pathways of interest and generate hypotheses in the context of genome-scale functional maps of human tissues. The precise actions of genes are frequently dependent on their tissue context, yet direct assay of tissue-specific protein function and interactions remains infeasible in many normal human tissues and cell-types. With GIANT2, researchers can explore predicted tissue-specific functional roles of genes and reveal changes in those roles across tissues, all through interactive multi-network visualizations and analyses. Additionally, the NetWAS approach available through the server uses tissue-specific/cell-type networks predicted by GIANT2 to re-prioritize statistical associations from GWAS studies and identify disease-associated genes. GIANT2 predicts tissue-specific interactions by integrating diverse functional genomics data from now over 61 400 experiments for 283 diverse tissues and cell-types. GIANT2 does not require any registration or installation and is freely available for use at http://giant-v2.princeton.edu.

## INTRODUCTION

Tissue and cell type specificity are critical aspects of complex human disease. From impaired insulin signaling in diabetes (1,2) to neuronal loss in Parkinson's disease (3–5), understanding tissue- and cell-lineage specific processes is necessary in elucidating disease pathophysiology and disease-gene relationships. However, direct assay of tissue-specific function is highly challenging and in many human tissues and cell-types remains infeasible. Yet mapping these tissue-specific interactions is key to understanding pathway action in different tissues and their role in the manifestation of human disease.

Many resources collect and provide access to rich functional genomics data. For example, resources such as BioGRID (6) and Reactome (7) curate interaction data for querying and visualization. These data, however, represent global pathway function and cannot distinguish the tissue-specific actions of genes. Some resources such as Gene Expression Tissue Project (GTEx) (8) enable access to a rich collection of tissue expression profiles, and more broadly, NCBI GEO (9) provides search of thousands of gene expression experiments. Altogether, these resources provide measurements of genes’ cellular activity, however they must be integrated to understand the precise functions of genes, particularly in a multi-cellular context. Successful methods by us (10–12) and others (13,14) can integrate these functional genomics data to predict functional interactions in human, many of which are accessible through a web server (13–15). However, these predictions lack tissue-specificity and none of them capture tissue and cell-type specific gene function, critical to understanding the complex and context-specific action of genes. Further, none of these resources can leverage tissue-specific interactions to aid researchers in the analysis of quantitative genetics data.

GIANT (Genome-wide Analysis of gene Networks in Tissues), introduced in 2015 (16), is a prediction server for human tissue-specific gene interactions that enables biomedical researchers to interrogate tissue-specific action through multi-network visualizations and analyses. Researchers can interact with GIANT by submitting individual genes or gene sets of interest for real-time integration of thousands of functional genomics experiments to predict tissue-specific interactions relevant to these genes and related processes. GIANT will return dynamic, interactive visualizations of predicted tissue-specific maps of the queried genes and tissues, and network-driven predictions of gene function and disease association. Additionally, GIANT allows users to run NetWAS (16), a machine-learning based method that leverages tissue-specific interactions to reprioritize genome-wide association data and identify disease-associated genes. NetWAS analysis is performed entirely server-side, requiring no software installation or specific computational resources from the users. In addition to user-friendly, interactive visualizations, all predicted networks and user's NetWAS results are available for download.

The probabilistic model used in GIANT2 infers tissue-specific interactions from large data compendia by simultaneously extracting functional and tissue or cell-type specific signals, and has been extensively evaluated in our previous work (16). We showed that GIANT networks could predict the lineage-specific response to IL-1B stimulation in blood vessel, which was then experimentally confirmed. This result was not exclusive to blood vessel—we additionally showed that GIANT made accurate predictions for tissue- and cell-lineage-specific response post IL-1B simulation for all tissues and cell-types for which public data were available. Furthermore, GIANT could capture the changing functional roles of LEF1 across tissues, and map the disease-disease associations of Parkinson's disease. We introduced NetWAS, a method to effectively re-prioritize statistical associations from a GWAS study with predicted tissue-specific interactions. With this approach, GIANT re-prioritized associations from a hypertension study, correctly identifying known hypertension genes, disease-related processes, and drug targets (without any prior knowledge of disease) and identified many candidate disease genes. GIANT has been continuously developed since original publication and here we describe the major updates to the server.

## SYSTEM DESCRIPTION AND UPDATES

**Figure 1.**
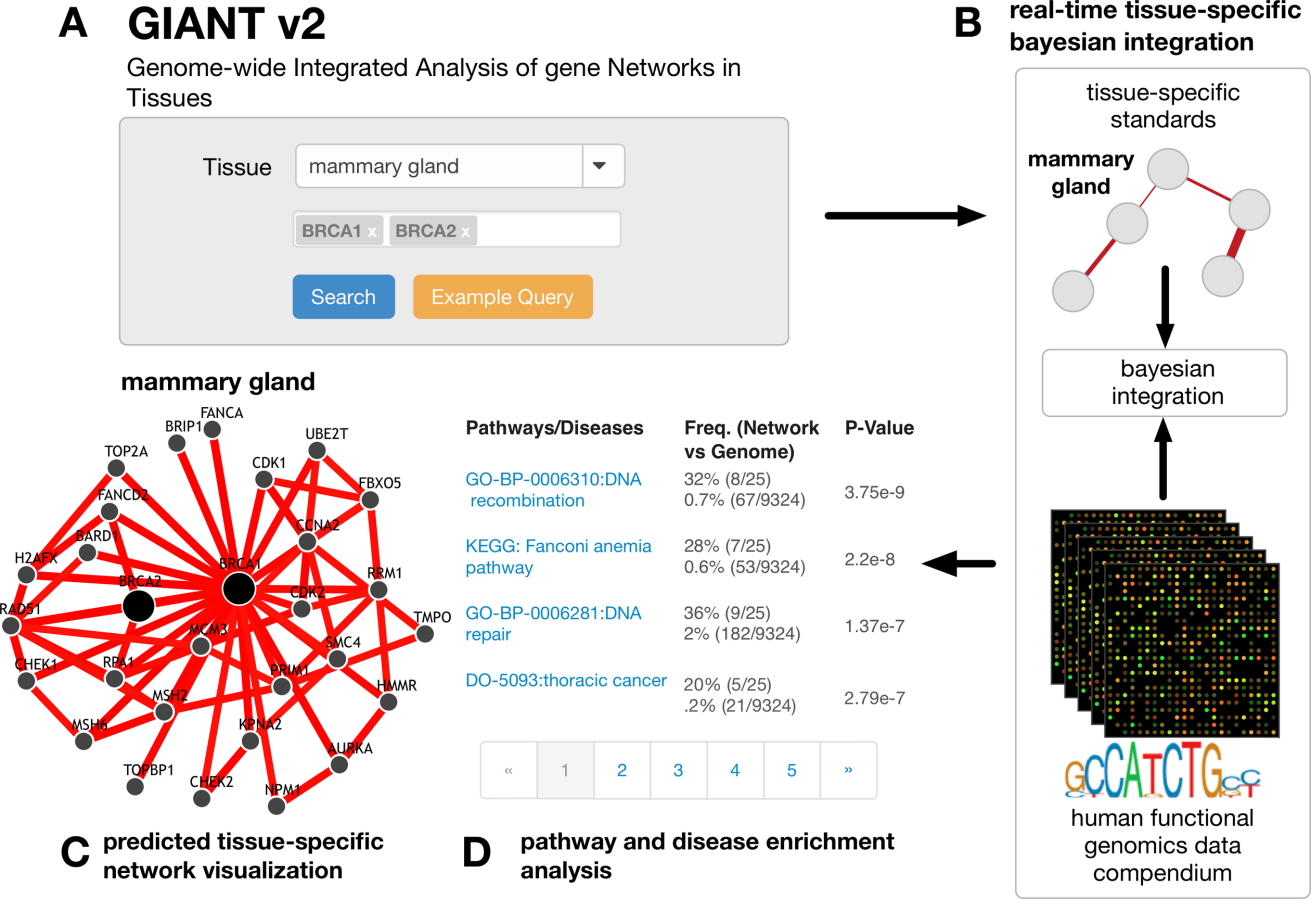
A schematic of the GIANT tissue-specific interaction prediction server. (**A**) GIANT is queried with two genes BRCA1 and BRCA2 in the *mammary gland* tissue. For optimal performance, we suggest that users query less than ten genes, in at most four tissues. The server response time increases with the number of queried genes and tissues (typically 2 seconds per gene/tissue). (**B**) GIANT integrates thousands of datasets from the human data compendium on-the-fly and predicts interactions to BRCA1 and BRCA2 with pre-computed tissue-specific Bayesian models. (**C**) The predicted interactions to BRCA1 and BRCA2 are shown as a network visualization where edges are predicted posterior probabilities of two genes functionally interacting in mammary gland. (**D**) Additional pathway and disease enrichment analysis of the displayed network is available to the user.

A GIANT prediction starts with a set of genes and one or more tissues of interest specified by the user (Figure 1A). The server predicts the likelihood of functional relationships between these genes and to all other genes in the human genome, for each of the queried tissues, by probabilistically integrating thousands of genome-scale experiments in a tissue-specific manner (Figure 1B). The results are presented to the user as a gene network for each queried tissue, with posterior probabilities of functional relationships for the genes of interest specific to that tissue (Figure 1C). These predictions can reveal the tissue-specific pathway partners or functional roles of the genes of interest. GIANT server provides extensive user-friendly visualizations enabling the user to seamlessly explore these predicted networks. Users can adjust the visualization to suit their biological question by filtering interactions by confidence level, or limiting the network to the highest-connected genes. Additionally, GIANT provides dynamic gene enrichment analysis of the queried network (Figure 1D). Gene Ontology (GO) biological process (17), Kyoto Encyclopedia of Genes and Genomes (KEGG) (18) pathway and Online Mendelian Inheritance in Man (OMIM) (19) disease-gene enrichments are calculated in real-time as users adjust the visualized network. These version-controlled gene sets are downloaded from the Tribe web server (bioRxiv: https://doi.org/10.1101/055913) and made available on GIANT. These analyses aid interpretation of large gene sets, which are often the outcome of a high-throughput experiment, and help generate hypotheses for experimental follow-up.

A key feature of GIANT networks is the ability to delineate the tissue-specific changes of multifunctional genes. The GIANT web server enables this feature with advanced multi-network visualization. When users query GIANT with multiple tissues, gene interactions for each tissue are simultaneously predicted and displayed as separate networks with a coordinated layout. Genes that are shared across tissues are both highlighted visually and positioned similarly in their respective views. Interactions with a tissue-network are mirrored across network views. Altogether, these features are designed to aid interpretation of genes’ changing interaction partners, and thus biological function, across tissues and cell-types.

GIANT also uses tissue-specific networks to make predictions that provide novel hypotheses related to human disease. NetWAS, in conjunction with tissue-specific/cell-type networks predicted by GIANT, effectively re-prioritizes statistical associations from distinct GWAS to identify disease-associated genes. Biologists can submit a GWAS result file, select a tissue relevant to the studied phenotype, and run NetWAS on the GIANT server.

The newest version of GIANT doubles the number of tissues and cell-types for which it can make on-the-fly functional network predictions to 283, including networks for 105 specific cell types (compared to 144 total networks and 23 specific cell types in the original GIANT release). Many of these cell types (and even tissues) are very challenging or impossible to assay experimentally in humans, with predictions from the GIANT server providing the only systems-level molecular coverage. We have carefully collected tissue-gene gold standards from established genomics resources (GTEx (8) and FANTOM5 (20)), improving both gene and tissue/cell-type coverage as compared to prior sources (21). These tissue-expression profiles are used to define tissue-gene relationships and to weight gene pairs by tissue-specificity during model training (See Supplemental Methods). We have also adopted a more uniform and well-maintained ontology of tissues and cell types (UBERON (22) and Cell Ontology (22)). This resulted in both a substantial increase in training data for each tissue, and in the total tissues and cell-types for which we could confidently predict interactions. Furthermore, the 283 GIANT2 network predictions are made based on over 61 400 experiments from 24 930 publications, spanning diverse data types (e.g. mRNA expression and protein-protein interaction data). This is a 60% increase in the experimental coverage compared to GIANT’s original release. The updated web-server has been available and running for a year.

## EXAMPLE USE CASE: MULTI-TISSUE ANALYSIS

With GIANT2, biologists can interrogate gene function in 283 diverse tissues and cell-types with multi-network visualizations and analyses. GIANT can reveal the changing roles of multifunctional genes by comparing the predicted tissue-specific interactions, the enriched biological processes, and gene-disease associations across tissues.

**Figure 2.**
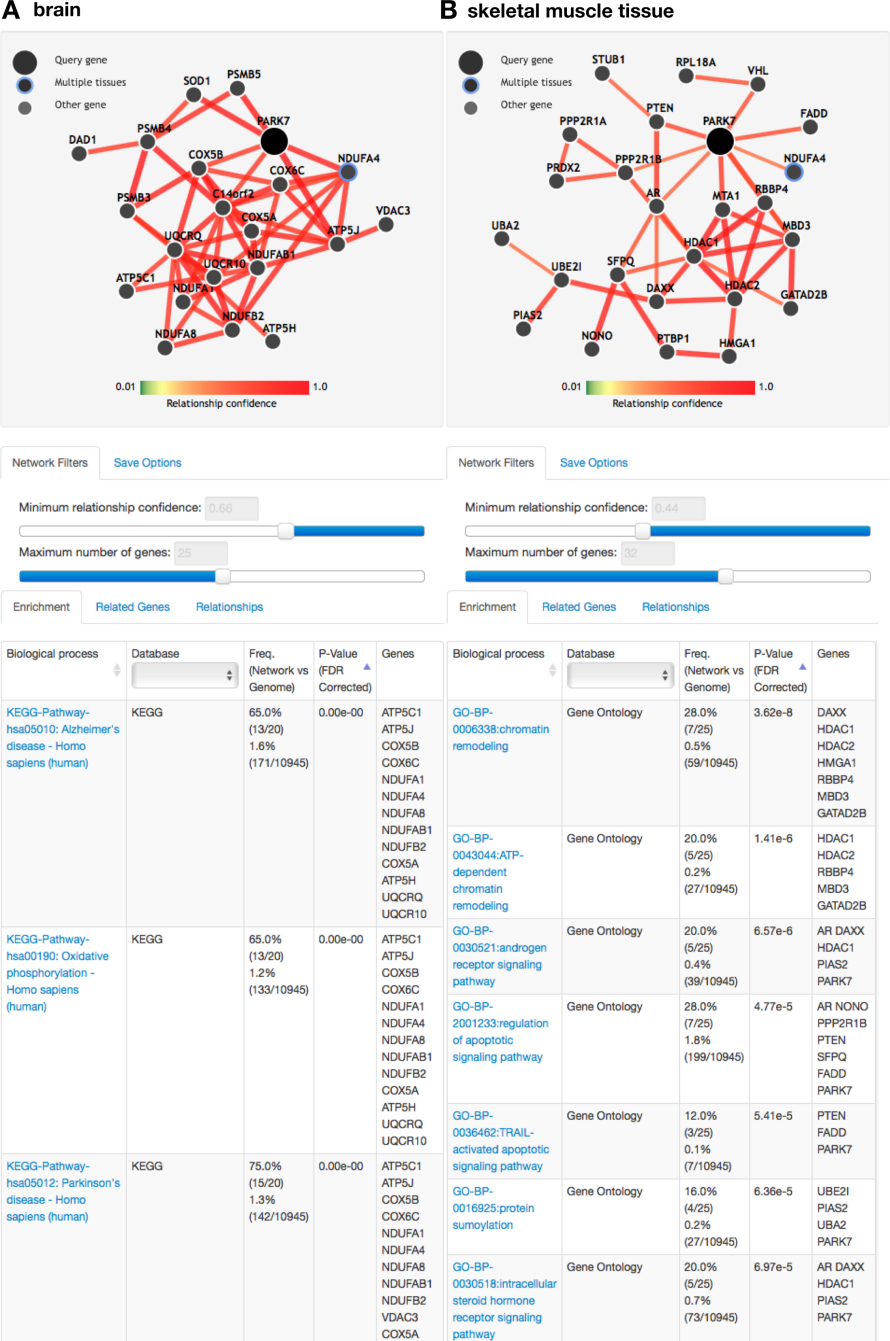
GIANT multi-network visualization. (**A**) Network showing PARK7 interactions in *brain*. The genes with the highest confidence interactions to PARK7 in *brain* are highly enriched for Parkinson's disease associated genes, among others. (**B**) In the skeletal muscle tissue network, PARK7 and its neighbors are enriched for androgen receptor signaling pathways.

In Figure 2, the user queries the multifunctional gene PARK7 in two tissues: *brain* and *skeletal muscle tissue*. GIANT returns predicted tissue-specific interactions to PARK7 in the two tissues and displays them as separate network views. The interactions are visualized with a coordinated layout where common genes have the same position in their respective network visualizations. The PARK7 interaction partners in brain and skeletal muscle tissue are considerably different, reflecting the different functional roles of PARK7. Notably, in the brain network (Figure 2A), PARK7 and its partners are significantly enriched for genes involved in Parkinson's disease (PD), consistent with PARK7’s known role in familial PD (23). In skeletal muscle tissue (Figure 2B), PARK7 interaction partners are highly enriched for ‘androgen receptor signaling pathway’. Human PARK7 has been previously established as a regulator of androgen receptor (24,25), whose signaling contributes to muscle mass maintenance (26), and PARK7 orthologs have been specifically linked to muscle hypertrophy (27). Thus, as shown with this example, GIANT-predicted tissue-specific networks are able to distinguish the distinct functional roles of PARK7, revealed through differences in predicted interactions. These predictions might help biomedical researchers studying PARK7 as a therapeutic target understand its tissue-specific pleiotropic effects.

## EXAMPLE USE CASE: NETWORK-GUIDED GWAS

Most complex diseases have tissue-specific origins and manifestations. With NetWAS, the tissue-specific/cell-type interactions captured in GIANT networks are used to re-prioritize results from a genome-wide association study of interest to the user. NetWAS is premised on the idea that top GWAS associations are enriched with disease-relevant genes, even if they fall below statistical significance (16). By learning the connectivity patterns of these top genes in relevant tissue networks, NetWAS can further enrich for phenotype-associated genes in a genome-wide re-ranking of the GWAS.

NetWAS trains a support-vector-machine (SVM), where the features of the SVM are interactions between genes in the selected tissue networks, positive labels are genes whose *P*-value fall below a selected cutoff, and negative labels are random genes above the cutoff. The SVM classifies—with five-fold cross-validation—all genes in the genome based on the tissue-specific interactions of the top GWAS genes. Note that no prior disease knowledge is used in this process - all disease signal is extracted from the GWAS study. Thus, NetWAS is discovery driven, where the GWAS itself is used to identify connectivity patterns rather than limited and potentially biased prior disease knowledge.

**Figure 3.**
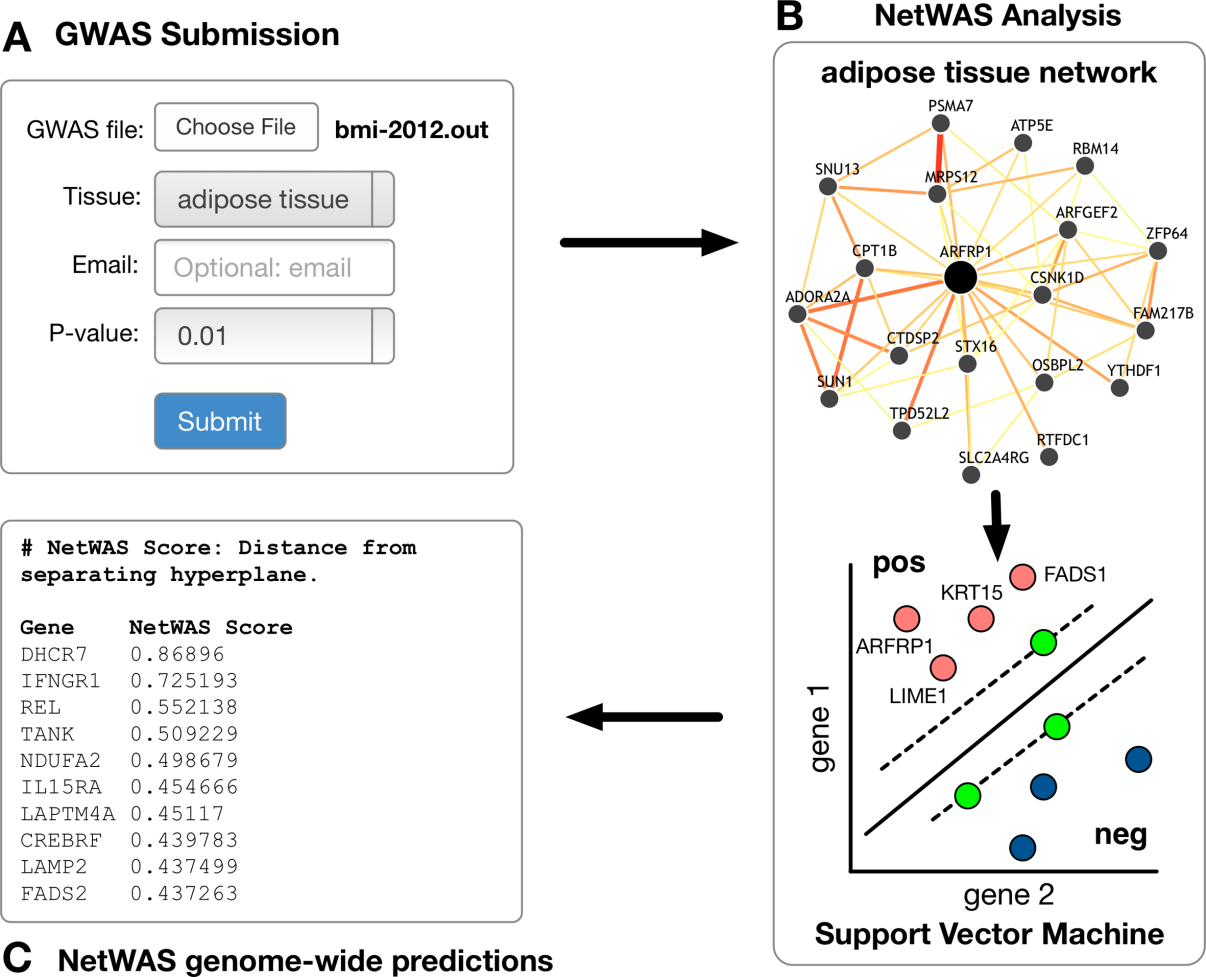
A schematic of a NetWAS analysis. (**A**) A user submits a BMI GWAS result file consisting of gene-wise *P*-values and selects ‘adipose tissue’ as a relevant tissue and a *P*-value cutoff of 0.01. (**B**) NetWAS builds an SVM where features are the predicted tissue-specific interactions in adipose tissue, positive labels are genes in the BMI GWAS whose *P*-value is less than 0.01 and negative labels are random genes whose *P*-value is above 0.01. (**C**) NetWAS results are a re-ranking of all genes in the genome. The NetWAS score is the direct output of the SVM (i.e. distance to the separating hyperplane). Higher (positive) scores indicate that a gene is more likely to be associated with the studied trait. The results can be emailed to the user and are available directly through GIANT through a unique result-specific URL.

Figure 3 shows the NetWAS workflow for a GWAS of Body Mass Index (BMI) (28) with adipose tissue. A researcher with a GWAS result (bmi-2012.out) uploads her result file of gene association *P*-values using the GIANT (Figure 3A) web form. GIANT supports many file formats of commonly used tools that pool SNP associations to gene-wise *P*-values (29)—a required step before running NetWAS. The user selects two options taking into account her particular GWAS result: (i) a *P*-value cutoff used to select ‘top’ genes for training (the default is 0.01, which has been successfully applied in many NetWAS analyses (16)) and (ii) a tissue/cell-type relevant to the studied phenotype (adipose tissue). Upon submission, NetWAS is run on GIANT servers (Figure 3B) and does not require software installation or dedicated computational resources by the user. The result is a genome-wide re-ranking of genes driven by their network similarity to the top GWAS genes (Figure 3C). This re-ranking has been shown (16) to improve disease association signal over the original GWAS in this BMI study (28), and many others (30,31).

## SUMMARY

GIANT is a dynamic, interactive web server that offers biologists a diverse collection of tools to answer experimental questions in the context of human tissue-specific functional maps. GIANT integrates thousands of genomics datasets to predict gene interactions in 283 tissues and cell-types, and enables re-analysis of quantitative genetics data through NetWAS. These tools are accessible to biomedical researchers through a user-friendly interface with flexible visualizations. Importantly, the tools and analyses in GIANT are data-driven and reach beyond existing, curated biological knowledge. Thus, GIANT can complement the tools of modern biologists to interpret and guide experiments involving tissue-specific gene action.

## Supplementary Material

Supplementary Data
