# Supplementary material for: GIANT 2.0: genome-scale integrated analysis of gene networks in tissues
Source: Nucleic Acids Res. 2018 May 25;46(Web Server issue):W65–70. doi: 10.1093/nar/gky408 (PMC6030827; doi:10.1093/nar/gky408)
Supplement: Supplementary Data [file gky408_supplemental_files.pdf]

Supplemental Methods

The methods in this GIANT update follow as previously described in the 2015 paper (1), except where noted.

Data download and processing

We collected 1540 genome-scale data sets including 61,400 conditions from an estimated 24,900 publications. Interaction data were downloaded from BioGRID (2), IntAct (3), MINT (4) and MIPs (5). Shared transcription factor regulation was estimated from JASPAR (6) binding motifs. Chemical and genetic perturbation (c2:CGP) and microRNA target (c3:MIR) profiles were downloaded from the Molecular Signatures Database (MSigDB) (7). All datasets were processed as previously described (1) and those methods are reproduced here with updated summary counts:

BioGRID edges were discretized into five bins, labeled 0 to 4, where the bin number reflected the number of experiments supporting the interaction. For the remaining databases, edges were discretized into the presence or absence of an interaction.

To estimate shared transcription factor regulation, binding motifs were downloaded from JASPAR. Genes were scored for the presence of transcription factor binding sites using the MEME software suite (8). FIMO (9) was used to scan for each transcription factor profile within the 1-kb sequence upstream of each gene. Motif matches were treated as binary scores (present if $P < 0.001$). The final score for each gene pair was obtained by calculating the Pearson correlation between the motif association vectors for the genes.

Chemical and genetic perturbation (c2:CGP) and microRNA target (c3:MIR) profiles were downloaded from the Molecular Signatures Database (MSigDB) (7). Each gene pair's score was the sum of shared profiles weighted by the specificity of each profile (1/len(genes)). The resulting scores were converted to *z* scores and discretized into bins ((−infinity, −1.5), [−1.5, −0.5), [–0.5, 0.5), [0.5, 1.5), [1.5, 2.5), [2.5, 3.5), [3.5, 4.5), [4.5, infinity)).

We downloaded all gene expression data sets from NCBI's Gene Expression Omnibus (GEO) (10) and collapsed duplicate samples. GEO contains 1533 human data sets. Genes with more than 30% of values missing were removed, and remaining missing values were imputed using ten neighbors. Non-log-transformed data sets were log transformed. Expression measurements were summarized to Entrez identifiers, and duplicate identifiers were merged. The Pearson correlation was calculated for each gene pair, normalized with Fisher's z transform, mean subtracted and divided by the standard deviation. The resulting z scores were discretized into bins ((−infinity, −1.5), [–1.5, −0.5), [–0.5, 0.5), [0.5, 1.5), [1.5, 2.5), [2.5, 3.5), [3.5, infinity)).

Hierarchically aware gold standard construction

Functional knowledge extraction. We constructed a tissue-naive functional relationship gold standard as described previously but with updated GO annotations (11). We processed experimentally derived gene annotations (GO evidence codes: EXP, IDA, IPI, IMP, IGI and IEP) from a set of 618 expert-selected GO biological process terms. To increase the coverage of functional interactions, we transferred experimentally confirmed mouse GO annotations to human functional analogs identified by FKT (12), a high-specificity annotation transfer method, for the 592 GO terms with mouse annotations. This resulted in a tissue-naive gold standard of 1,393,224 functionally related gene pairs (positive examples) and 16,281,559 potentially unrelated pairs (negative examples).

Ontology-aware gene-tissue annotations. Gene-to-tissue annotations were derived from GTEx (13) and FANTOM5 (14) RNA-seq data. Expression data, in transcripts per million (TPM) units, from both resources were quantile normalized to enable cross-sample comparisons. Samples were mapped to tissue and cell-type terms in UBERON (15) and Cell Ontology and propagated along a shared hierarchy. Genes were assigned to tissues (designated as 'tissue-expressed') given the following rules:

1. A gene was declared in a *sample* as:
   - 'ON' if its TPM ≥ 6 and TPM ≥ *gene_median*
   - 'OFF' if its TPM ≤ 1 and TPM < *gene_median*
2. A gene was declared in a *tissue* as:
   - 'not-expressed' if the gene is 'OFF' in ≥ 3 samples and 'ON' in ≤ 1 sample
   - 'tissue-expressed' if 'ON' in ≥ 3 samples and 'not-expressed' ≥ 2 unrelated tissues (based on the tissue/cell-type ontology)

where *gene_median* is the median TPM of a gene across all samples.

Integration of tissue-specific and functional knowledge. We combined the above gene-to-tissue annotations with the tissue-naive functional gold standard to construct a hierarchical tissue-specific knowledgebase. We labeled each gene pair (positive or negative) in the tissue-naive functional relationship standard as specifically coexpressed in a tissue if both genes were designated as tissue-expressed (T, T).

After labeling specifically coexpressed gene pairs (edges) across all tissues, we considered four classes of edges—C1, C2, C3 and C4—to constitute each tissue standard.

C1: positive functional edges between genes specifically co-expressed in the tissue [T–T].
C2: positive functional edges between a gene expressed in the tissue and another specifically expressed in an unrelated tissue [T–T′].
C3: negative functional edges between genes specifically co-expressed in the tissue [T–T].
C4: negative functional edges between one gene expressed in the tissue and another specifically expressed in an unrelated tissue [T–T′].

Among the four tissue classes, C1 represented tissue-specific functional relationships. To identify tissue-specific relationships, we constructed a specific gold standard for each tissue by labeling edges in C1 as positives and edges in the other classes as negatives. Because C3 is defined on the basis of tissue-expressed genes and C2 and C4 are defined on the basis of non-expressed genes, the number of edges in these classes varied across tissues according to how specific (cell type, tissue, organ or system), well studied (or easily studied) and well curated (literature bias) they are. To construct comparable networks across tissues, we used a negative set composed of equal proportions of edges from C2, C3 and C4.

Tissue-specific weighting. We calculated a weight for each C1 edge corresponding to its tissue-specificity. For every gene represented in FANTOM5 (14) and GTEx (13) (as processed above), we calculated tissue expression as the median TPM of the gene across all samples corresponding to the tissue. We calculated tissue-specificity as the z-score of a gene's expression in a tissue (x_i) compared to its mean (u) and standard deviation expression across all non-related tissues - defined as tissues that do not share the same tissue system (e.g. for brain, non-related tissue were all tissues not part of the nervous system).

$$z = \frac{x_i - \mu}{\sigma}$$

$$c_{g1g2} = \max(z_{g1}, z_{g2}, 0)$$

For a positive edge with incident genes g_1 and g_2, c_{g1g2} is the number of counts the edge will contribute to the conditional probability table during the learning phase of the naive bayes classifier. Note that gene pairs whose constituent genes are not specifically expressed (both gene z-scores are less than 0) will effectively be excluded during learning.

Data Integration. We constructed functional networks from genome-scale data by performing a weighted tissue-specific Bayesian integration. We trained one naive Bayesian classifier for each tissue using the tissue-specific standards described above, where each positive edge was additionally weighted by the tissue-specific expression of the incident genes, as described above.

In each case, we constructed a class node, i.e., the presence or absence of a functional relationship between a pair of genes that is conditioned on nodes for each data set. For large-scale genomics data sets, the assumption of conditional independence required for a naive Bayes classifier is often not met, so we calculated and corrected for non-biological conditional dependency (12).

Each tissue model trained on the hierarchy-aware tissue-specific knowledge was used to make genome-wide predictions by estimating the probability of tissue-specific functional interaction between all pairs of genes. We also estimated the probability of global functional interactions for the tissue-naive network. We assigned a prior probability of a functional relationship of 0.1 for all models, allowing edge probabilities to be compared across tissues.

Network-based reprioritization of genome-wide association study.

NetWAS was implemented as previously described (1). We trained a support vector machine classifier using nominally significant (P < user defined cutoff) genes as positive examples and 10,000 randomly selected non-significant ($P \geq$ user defined cutoff) genes as negatives. The classifier was constructed using the chosen tissue network, where the features of the classifier were the edge weights of the labeled examples to all the genes in the network. Genes were re-ranked using their distance from the hyperplane.

Bayesian integration and NetWAS analysis was performed with the open-source C++ software, Sleipnir Library for Computational Functional Genomics (16), available at: http://libsleipnir.bitbucket.io/

1. Greene, C.S., Krishnan, A., Wong, A.K., Ricciotti, E., Zelaya, R.A., Himmelstein, D.S., Zhang, R., Hartmann, B.M., Zaslavsky, E., Sealfon, S.C. *et al.* (2015) Understanding multicellular function and disease with human tissue-specific networks. *Nature genetics*, **47**, 569-576.
2. Chatr-Aryamontri, A., Oughtred, R., Boucher, L., Rust, J., Chang, C., Kolas, N.K., O'Donnell, L., Oster, S., Theesfeld, C., Sellam, A. *et al.* (2017) The BioGRID interaction database: 2017 update. *Nucleic acids research*, **45**, D369-D379.
3. Kerrien, S., Aranda, B., Breuza, L., Bridge, A., Broackes-Carter, F., Chen, C., Duesbury, M., Dumousseau, M., Feuermann, M., Hinz, U. *et al.* (2012) The IntAct molecular interaction database in 2012. *Nucleic acids research*, **40**, D841-846.
4. Licata, L., Briganti, L., Peluso, D., Perfetto, L., Iannuccelli, M., Galeota, E., Sacco, F., Palma, A., Nardozza, A.P., Santonico, E. *et al.* (2012) MINT, the molecular interaction database: 2012 update. *Nucleic acids research*, **40**, D857-861.
5. Mewes, H.W., Frishman, D., Guldener, U., Mannhaupt, G., Mayer, K., Mokrejs, M., Morgenstern, B., Munsterkotter, M., Rudd, S. and Weil, B. (2002) MIPS: a database for genomes and protein sequences. *Nucleic acids research*, **30**, 31-34.
6. Portales-Casamar, E., Thongjuea, S., Kwon, A.T., Arenillas, D., Zhao, X., Valen, E., Yusuf, D., Lenhard, B., Wasserman, W.W. and Sandelin, A. (2010) JASPAR 2010: the greatly expanded open-access database of transcription factor binding profiles. *Nucleic acids research*, **38**, D105-110.
7. Subramanian, A., Tamayo, P., Mootha, V.K., Mukherjee, S., Ebert, B.L., Gillette, M.A., Paulovich, A., Pomeroy, S.L., Golub, T.R., Lander, E.S. *et al.* (2005) Gene set enrichment analysis: a knowledge-based approach for interpreting genome-wide expression profiles. *Proc Natl Acad Sci U S A*, **102**, 15545-15550.
8. Bailey, T.L., Boden, M., Buske, F.A., Frith, M., Grant, C.E., Clementi, L., Ren, J., Li, W.W. and Noble, W.S. (2009) MEME SUITE: tools for motif discovery and searching. *Nucleic acids research*, **37**, W202-208.
9. Grant, C.E., Bailey, T.L. and Noble, W.S. (2011) FIMO: scanning for occurrences of a given motif. *Bioinformatics (Oxford, England)*, **27**, 1017-1018.
10. Barrett, T., Wilhite, S.E., Ledoux, P., Evangelista, C., Kim, I.F., Tomashevsky, M., Marshall, K.A., Phillippy, K.H., Sherman, P.M., Holko, M. *et al.* (2013) NCBI GEO: archive for functional genomics data sets--update. *Nucleic acids research*, **41**, D991-995.
11. Ashburner, M., Ball, C.A., Blake, J.A., Botstein, D., Butler, H., Cherry, J.M., Davis, A.P., Dolinski, K., Dwight, S.S., Eppig, J.T. *et al.* (2000) Gene ontology: tool for the unification of biology. The Gene Ontology Consortium. *Nature genetics*, **25**, 25-29.
12. Park, C.Y., Wong, A.K., Greene, C.S., Rowland, J., Guan, Y., Bongo, L.A., Burdine, R.D. and Troyanskaya, O.G. (2013) Functional knowledge transfer for high-accuracy prediction of under-studied biological processes. *PLoS computational biology*, **9**, e1002957.
13. Consortium, G.T. (2013) The Genotype-Tissue Expression (GTEx) project. *Nature genetics*, **45**, 580-585.
14. Lizio, M., Harshbarger, J., Shimoji, H., Severin, J., Kasukawa, T., Sahin, S., Abugessaisa, I., Fukuda, S., Hori, F., Ishikawa-Kato, S. *et al.* (2015) Gateways to the FANTOM5 promoter level mammalian expression atlas. *Genome Biol*, **16**, 22.

15. Mungall, C.J., Torniai, C., Gkoutos, G.V., Lewis, S.E. and Haendel, M.A. (2012) Uberon, an integrative multi-species anatomy ontology. *Genome Biol*, **13**, R5.
16. Huttenhower, C., Schroeder, M., Chikina, M.D. and Troyanskaya, O.G. (2008) The Sleipnir library for computational functional genomics. *Bioinformatics (Oxford, England)*, **24**, 1559-1561.
